# Evaluation of the ability of fatty acid metabolism signature to predict response to neoadjuvant chemoradiotherapy and prognosis of patients with locally advanced rectal cancer

**DOI:** 10.3389/fimmu.2022.1050721

**Published:** 2022-11-24

**Authors:** Han Zhou, Yanping Chen, Yu Xiao, Qian Wu, Hui Li, Yi Li, Guangjian Su, Longfeng Ke, Junxin Wu, Jinluan Li

**Affiliations:** ^1^ Department of Radiation Oncology, Clinical Oncology School of Fujian Medical University, Fujian Cancer Hospital, Fuzhou, China; ^2^ Department of Clinical Pathology, Clinical Oncology School of Fujian Medical University, Fujian Cancer Hospital, Fuzhou, China; ^3^ Department of Clinical Laboratory, Clinical Oncology School of Fujian Medical University, Fujian Cancer Hospital, Fuzhou, China

**Keywords:** fatty acid metabolism, gut microbiome, metabolite, neoadjuvant chemoradiotherapy response, rectal cancer

## Abstract

Neoadjuvant chemoradiotherapy (nCRT) is widely used to treat patients with locally advanced rectal cancer (LARC), and treatment responses vary. Fatty acid metabolism (FAM) is closely associated with carcinogenesis and cancer progression. In this study, we investigated the vital role of FAM on the gut microbiome and metabolism in the context of cancer. We screened 34 disease-free survival (DFS)-related, FAM-related, and radiosensitivity-related genes based on the Gene Expression Omnibus database. Subsequently, we developed a five-gene FAM-related signature using the least absolute shrinkage and selection operator Cox regression model. The FAM-related signature was also validated in external validation from Fujian Cancer Hospital for predicting nCRT response, DFS, and overall survival (OS). Notably, patients with a low-risk score were associated with pathological complete response and better DFS and OS outcomes. A comprehensive evaluation of the tumor microenvironment based on the FAM-related signature revealed that patients with high-risk scores were closely associated with activating type I interferon response and inflammation-promoting functions. In conclusion, our findings indicate the potential ability of FAM to predict nCRT response and the prognosis of DFS and OS in patients with LARC.

## Introduction

Colorectal cancer (CRC) is one of the three most common cancers and the second leading cause of cancer-related deaths worldwide (1). Rectal cancer accounts for approximately 30% of all newly diagnosed CRC cases (2). Neoadjuvant chemoradiotherapy (nCRT) followed by total mesorectal excision is the standard treatment modality for patients with locally advanced rectal cancer (LARC) (T3, T4, or N+) (3). nCRT has been confirmed to be associated with better survival outcomes, especially in improving disease-free survival (DFS) rates (4, 5). Patients with pathological complete response (pCR) have been confirmed to have much better overall survival (OS) and DFS. However, treatment responses varied widely among patients. About 15%–30% of patients achieve pCR after nCRT (6, 7). Hence, it is crucial to identify potential biomarkers to predict treatment response and prognosis in patients with rectal cancer who undergo nCRT.

Microbiomes and metabolites have been recognized as indispensable cancer hallmarks (8, 9). The gut microbiome is known to be related to tumor development, especially in digestive system tumors (10, 11). Metabolic reprogramming also plays an important role in cancer development (12–14). In addition, gut microbiome dysbiosis and metabolic disorders are associated with the development of CRC (15). The gut microbiome and metabolites can be used to predict treatment response to radiotherapy, chemotherapy, and immunotherapy (16, 17). In addition, the gut microbiome and metabolites have been shown to be useful in predicting nCRT response in patients with LARC (18, 19). However, little is known about the mechanisms by which the gut microbiome and metabolites influence radiotherapy response in rectal cancer (20).

Fatty acid metabolism (FAM) has been the focus of related research because of its close relationship with carcinogenesis and cancer progression (21). Fatty acids (FAs) are a principal structural component of the human body. They are also vital secondary messengers and materials for energy production (22). FAM has been confirmed to be associated with sensitivity to chemotherapy, radiotherapy, and targeted therapy in cancers (19, 23). Given the important role of FAM, therapies targeting FAM are of great concern. Previous studies have shown the potential ability of FAM-related genes to guide prognosis in CRC (24, 25). However, evidence of FAM-related genes predicting treatment response in patients with rectal cancer is lacking. Therefore, further exploration of the relationship between FAM-related genes and clinicopathological characteristics in patients with rectal cancer treated with nCRT would be helpful in developing personalized regimens and management.

In this study, we first analyzed the potential function of the gut microbiome and its metabolites in rectal cancer patients with different nCRT responses. Additionally, we established a reliable signature for FAM-related genes. We fully evaluated its ability to predict survival outcomes and treatment responses in rectal cancer patients treated with nCRT based on the Gene Expression Omnibus (GEO) database. The findings of this study provide new insights that may be used to personalize the treatment of patients with rectal cancer who have undergone nCRT.

## Materials and methods

### Study participants

**Figure 1:**
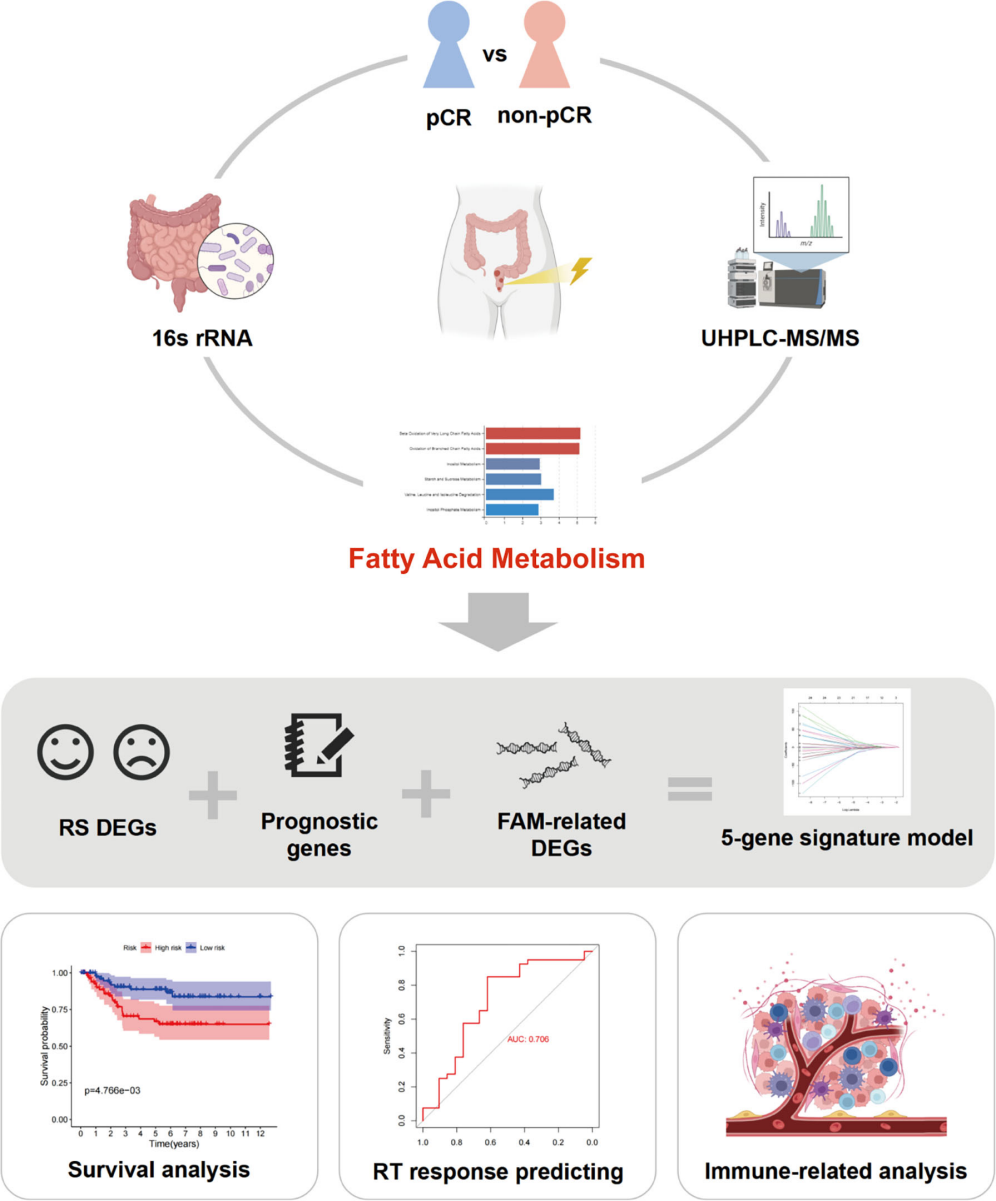
Workflow of this study. pCR, pathological complete response; UHPLC-MS/MS, ultra-high performance liquid chromatography–tandem mass spectrometry; RS, radiation sensitivity; FAM, fatty acid metabolism; RT, radiotherapy.

The procedure used in this study is shown in **Figure 1**. This study was approved by the Ethics Committee of the Fujian Cancer Hospital (No. K2017-082-01). Between September 2020 and September 2021, 42 patients with newly diagnosed rectal cancer were administered nCRT before surgery at Fujian Cancer Hospital. The inclusion criteria were 1) pathologically confirmed rectal adenocarcinoma; 2) clinical T3, T4, and/or N+ without distant metastasis; 3) long-course nCRT comprising 50 Gy in 25 fractions and concurrent oral capecitabine chemotherapy; 4) surgery 6–8 weeks after nCRT; 5) Fujian Province resident for >10 years. The exclusion criteria were as follows: 1) patients with metabolic diseases; 2) incomplete clinical information; 3) treatment interruption or did not accept nCRT before surgery; 4) antibiotic or steroid therapy within 6 months before nCRT.

The histopathological responses to nCRT were classified according to the American Joint Committee on Cancer tumor regression grade (TRG) system, which is considered to be the most accurate (26). Patients with TRG grade 0 (no residual tumor cells) were classified as pCR, whereas patients with TRG grades 1–3 were classified as non-pCR. These specimens were examined by two experienced clinical pathologists.

### 16S rRNA gene sequencing and bioinformatics analysis

The V3–V4 region of the rRNA gene was amplified using primers 341F and 806R (27). The “DADA2” package was used to convert the paired-end FASTQ files. Raw data were processed using Quantitative Insights Into Microbial Ecology 2 (QIIME2, v. 2021.11). Representative amplicon sequence variant (ASV) sequences were classified into organisms using a naive Bayesian model and the RDP classifier (v. 2.2), according to the SILVA database (v. 132).

The abundance statistics for each taxon were visualized using Krona (v. 2.6). Venn analysis was performed using the R project “VennDiagram” package. The biomarker features were screened using linear discriminant analysis effect size (LEfSe) software (v. 1.0). Kyoto Encyclopedia of Genes and Genomes (KEGG) pathway analyses of ASVs were performed using the Phylogenetic Investigation of Communities by Reconstruction of Unobserved States 2 (PICRUSt2) Tool (v. 2.1.4). Wilcoxon rank sum tests were performed in the R package “vegan”.

### Ultra-high-performance liquid chromatography–tandem mass spectrometry analysis and statistical metabolism analyses

Ultra-high-performance liquid chromatography–tandem mass spectrometry (UHPLC-MS/MS) analyses were performed using a Vanquish UHPLC System (Thermo Fisher Scientific, Waltham, MA, USA) coupled with an Orbitrap Q Exactive™ HF-X mass spectrometer (Thermo Fisher Scientific). The peaks were then matched with the mzCloud (https://www.mzcloud.org/), mz Vault (Thermo Fisher Scientific), and Masslist databases (www.maldi-msi.org/mass) to obtain accurate qualitative and quantitative results.

A partial least-squares discriminant analysis (PLS-DA) was conducted with the “ropls” package in R. Variable importance in projection (VIP) based on PLS-DA was used to rank candidate metabolites (p< 0.05, t-test; VIP ≥ 1). Metabolite set enrichment analysis (MSEA) was used to evaluate pathway overrepresentation using the MetaboAnalyst module with the R package “MSEAp”.

### Date source

Gene expression and clinicopathological information were downloaded from the GEO database (https://www.ncbi.nlm.nih.gov/geo/). Two GEO cohorts (GSE56699 and GSE87211) were used in our study. The RNA sequencing data of two cohorts were corrected for batch effects by using the R package “sva”. A total of 158 FAM-related genes were obtained from the Molecular Signatures Database (MSigDB) (http://www.broad.mit.edu/gsea/msigdb/, **Supplementary Table 1**). In addition, 82 frozen cancer samples of patients with LARC at Fujian Cancer Hospital (FJCH) between June 2016 to June 2021 were used for external validation. All the patients received nCRT and radical surgery. The clinical information of the GEO validation sets and FJCH set is detailed in **Supplementary Table 2**.

### Single-sample gene set enrichment analysis

Single-sample gene set enrichment analysis (ssGSEA) was performed using the R package “GSVA” to calculate the pathway activity of “Hallmark_Fatty_Acid_Metabolism”. The patients were then divided into high and low pathway activities based on the median of all patients’ pathway activity.

### Identification of differentially expressed genes

FAM-related differentially expressed genes (DEGs) of patients with high and low pathway activities were screened with an adjusted p-value of<0.05 by using the R package “limma”. The same method was used to identify the radiosensitive (RS) DEGs between nCRT-sensitive and nCRT-resistant patients. Univariate Cox regression analysis was performed to select prognosis-related DEGs based on DFS by applying the Kaplan–Meier R package “survival” with a p-value of <0.05.

### Development of the fatty acid metabolism-related signature

The intersection of FAM-related DEGs, RS DEGs, and prognosis-related DEGs yielded candidate FAM-related genes. To avoid overfitting, the least absolute shrinkage and selection operator (LASSO) Cox regression algorithm was applied using the R package “glmnet”. Finally, the risk score of FAM-related genes was calculated using the following formula:


$$Risk\;score=\sum_{i-1}^{n}\left(Coefficient_{i}\times Expression_{i}\right)$$

The patients were divided into high-risk and low-risk groups based on the median values. A heatmap of the model-related genes was generated for the two cohorts.

### Assessment of fatty acid metabolism-related signature

FAM-related signatures were divided into two cohorts (GSE56699 and GSE87211). Patients in both cohorts were divided into high- and low-risk groups based on their FAM-related signature. Patients in both groups were then evaluated using the Kaplan–Meier survival analysis of DFS and OS by using the R package “survival”. The time-dependent receiver operating characteristic (ROC) curve was calculated using the R package “timeROC” to evaluate the predictive accuracy of the FAM-score prognostic model.

### Evaluation of fatty acid metabolism-related signature and clinicopathological features

Multivariate Cox regression analysis and Wilcoxon rank sum tests were performed to identify the relationship between risk scores and clinical features. The area under the curve (AUC) analysis was calculated by using the R package “pROC” to evaluate the accuracy of using risk scores in predicting nCRT response in patients with rectal cancer.

### Function enrichment analysis of fatty acid metabolism-related signature

Gene Ontology (GO) function enrichment analysis and KEGG function enrichment analysis were performed based on DEGs between high-risk and low-risk patients.

### Relationship between fatty acid metabolism-related signature with tumor microenvironment and immune-related analysis

The R package “GSVA” was applied to calculate the abundance of 28 immune-infiltrating tumor cell types in each patient. The Wilcoxon test was used to assess the differences between patients with high-risk and low-risk scores. The ESTIMATE algorithm was used to evaluate the immunity, tumor purity, and stromal scores of each patient. Furthermore, we analyzed the differential expression levels of immune checkpoints between high-risk score patients and low-risk score patients by applying the R package “limma”.

### Tissue samples and quantitative real-time polymerase chain reaction

Quantitative real-time polymerase chain reaction (qRT-PCR) was performed on 82 rectal cancer samples from FJCH. Total RNA was extracted from paraffin sections of tumor tissues using the E.Z.N.A. FFPE RNA Isolation Kit (R6954-01; Omega Bio-Tek, Doraville, GA, USA). The primer sequences are listed in **Supplementary Table 3**. Quantitative real-time PCR (qPCR) was used to determine RNA levels using SYBR Green (29139149001; Roche, Basel, Switzerland). RNA levels were normalized to those of β-actin. All the qRT-PCR analyses were performed in triplicates, and the average value was calculated by the Livak method.

### Statistical analyses

R (version 3.6.1) was used to perform statistical analyses in this study. Bioinformatics analyses were performed using Omicsmart, which is a real-time interactive online data analysis platform (http://www.omicsmart.com). Pearson’s χ^2^ test and Student’s t-test were used to compare normally distributed variables, whereas the Kruskal–Wallis test and Wilcoxon rank sum test were used to compare non-normally distributed variables. Statistical significance was set at p< 0.05.

## Results

### Relationship between baseline gut microbiome and neoadjuvant chemoradiotherapy response in patients with rectal cancer

**Figure 2:**
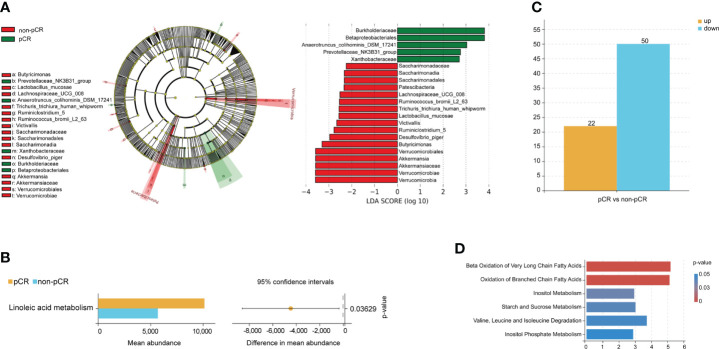
Gut microbiome and metabolism characteristics at the baseline associated with neoadjuvant chemoradiotherapy treatment response. **(A)** Differentially abundant taxa between pCR and non-pCR groups analyzed by LEfSe (Kruskal–Wallis test; p< 0.05; LDA > 2). **(B)** Differences in KEGG pathway analyses between pCR and non-pCR groups (p< 0.05). **(C)** The bar plots show upregulated and downregulated metabolites of patients with different nCRT responses. **(D)** MSEA of metabolites from each patient showed significantly enriched signaling pathways. nCRT, neoadjuvant chemoradiotherapy; pCR, pathological complete response; LEfSe, linear discriminant analysis (LDA) effect size; KEGG, Kyoto Encyclopedia of Genes and Genome; MSEA, metabolite set enrichment analysis.

Data from 14 patients were analyzed according to the inclusion and exclusion criteria. Detailed clinicopathological characteristics of the 14 patients are shown in **Supplementary Table 4**. There were 134 species included in the pCR group and 155 species in the non-pCR group (**Supplementary Figure 1B**). We first determined community composition at the species level in the top 10 microbiomes of the pCR and non-pCR groups (**Supplementary Figure 1A**). To further identify the significantly different gut microbiomes in patients with rectal cancer with different nCRT responses, we performed LEfSe with |log10 LDA| ≥ 2 (**Figure 2A**; **Supplementary Table 5**). The microbiome of the phylum Proteobacteria, including the class Betaproteobacteriales and the families Xanthobacteraceae and Burkholderiaceae, was enriched in the pCR group, which may be associated with treatment response.

Functional analysis was performed, and the results are shown in a streamgraph (**Supplementary Figure 1C**). We found that the functions of each patient were enriched in the metabolism-related pathways. Finally, the difference analysis between the pCR and non-pCR groups showed significant differences in linoleic acid metabolism, which is included in lipid and FAM (p< 0.05, **Figure 2B**).

### Characteristic of baseline metabolites of patients with rectal cancer with different neoadjuvant chemoradiotherapy responses

PLS-DA significantly segregated the patients into the pCR and non-pCR groups (**Supplementary Figure 1D**). We then screened the metabolites by combining the VIP scores based on PLS-DA and the p-values based on the t-test for the pCR and non-pCR groups. We found 72 metabolites with different abundance in patients with different nCRT responses at baseline (**Figure 2C**; **Supplementary Table 6**). Further functional enrichment analysis showed that different metabolites were primarily enriched in FAM (**Figure 2D**). Finally, propanoate and glycerol lipid metabolism were significantly different in patients with different nCRT responses (p< 0.05, **Supplementary Figure 1E**). Based on the analysis of the baseline gut microbiome and metabolites, we hypothesized that FAM may influence the response to nCRT in patients with rectal cancer.

### Identification of different expression genes and construction of fatty acid metabolism-related signatures

**Figure 3:**
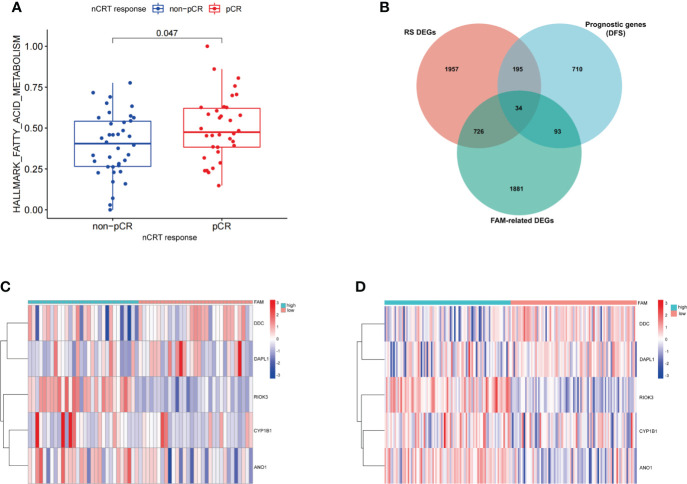
Construction of the FAM-related signature for patients with rectal cancer treated with neoadjuvant chemoradiotherapy. **(A)** The expression of FAM-related genes in relation to nCRT response. **(B)** Venn diagram identifying the intersection of genes among RS DEGs, FAM-related genes, and prognostic genes. **(C, D)** The expression levels of five FAM score-related genes in GEO database (GSE56699 and GSE87211). nCRT, neoadjuvant chemoradiotherapy; RS, radiation sensitivity; DEGs, differentially expressed genes; FAM, fatty acid metabolism; LASSO, least absolute shrinkage and selection operator.

We first scored 72 patients in GSE56699, based on 158 FAM-related genes (FAM-related genes). Patients who received pCR after nCRT had a higher score based on FAM-related genes than those who did not achieve pCR after nCRT (p< 0.05, **Figure 3A**). Once again, the results showed the vital role of FAM in influencing the treatment response in patients with rectal cancer. Thus, we divided the patients into two groups based on the median FAM-related genes score.

We identified 2,734 DEGs between the two groups with different FAM-related gene scores (**Supplementary Table 7**). Additionally, 2,912 RS DEGs were identified in patients with different nCRT responses (**Supplementary Table 8**), and 1,032 prognosis-related genes based on DFS were identified in patients from the GSE56699 dataset (**Supplementary Table 9**). Finally, 34 genes at the intersection of three parts of DEGs were recognized as potential candidate genes (**Figure 3B**; **Supplementary Table 10**). With the use of LASSO regression analysis, five genes (*CYP1B1*, *DDC*, *ANO1*, *DAPL1*, and *RIOK3*) were screened based on their minimum lambda values (**Supplementary Figure 2**, **Supplementary Table 11**). Among these five genes, two were protective genes (*DDC* and *DAPL1*) and three were risk-related genes (*CYP1B1*, *ANO1*, and *RIOK3*). Therefore, the FAM-related risk score was calculated using the following formula:


$$\begin{array}{l} FAM\;risk\;score=Expression\;of\;CYP1B1\times 0.2566+Expression\;of\\ DDC\times (-1.8227)+Expression\;of\;ANO1\times 1.2239+Expression\;of\\ DAPL1\times (-0.0843)+Expression\;of\;RIOK3\times 7.5436 \end{array}$$

According to the FAM-related risk score, patients with a score lower than the median risk score were classified into the low-risk group, whereas those with a score higher than the median risk score were classified into the high-risk group. The expression levels of the five genes are described in the heatmap of the GSE56699 and GSE87211 datasets (**Figures 3C, D**). *DDC* and *DAPL1* showed lower expression levels than *CYP1B1*, *ANO1*, and *RIOK3* in patients in the high-risk group.

### Prognostic analysis based on fatty acid metabolism-related signature

**Figure 4:**
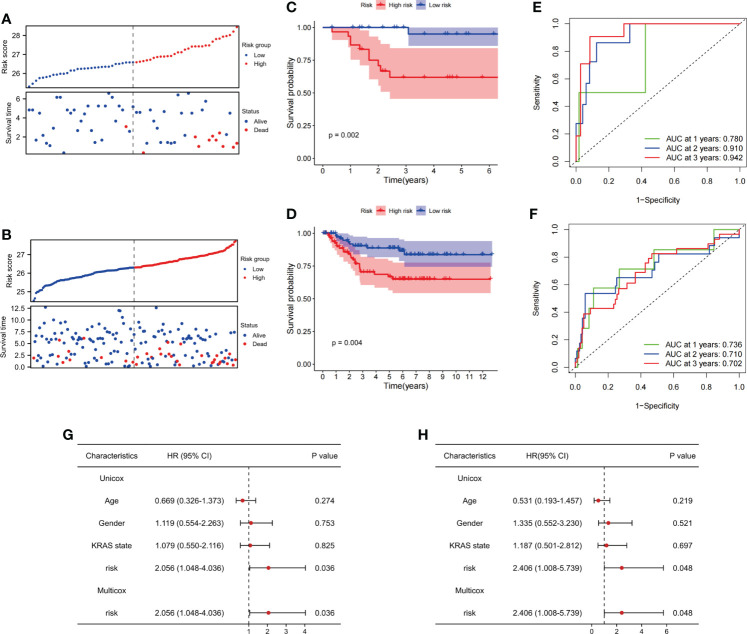
Evaluation of the ability of FAM-related signatures to predict prognosis in training and validation cohorts. **(A, B)** The association between DFS and FAM-related risk score in the training cohort (GSE56699) and the validation cohort (GSE87211). **(C, D)** Kaplan–Meier survival analyses of DFS between patients with high-risk scores and low-risk scores in the training and validation cohorts. **(E, F)** Time-dependent ROC curves used to evaluate the prognostic value of risk score in training and validation cohorts. **(G)** Univariate Cox analysis and multivariate Cox analysis of clinicopathological features and FAM-related signature in DFS. **(H)** Univariate Cox analysis and multivariate Cox analysis of clinicopathological features and FAM-related signature in OS. FAM, fatty acid metabolism; DFS, disease-free survival; ROC, receiver operating characteristic. OS, overall survival.

We evaluated the prognostic ability of FAM-related signatures in patients with rectal cancer from the training (GSE56699) and testing (GSE87211) datasets. All patients from the two datasets underwent nCRT. The distribution graph shows that the mortality rate of patients increased with an increase in the FAM-related risk score in the GSE56699 dataset (**Figure 4A**). The Kaplan–Meier survival curves revealed that patients in the low-risk group had significantly better DFS than those in the high-risk group (p< 0.05, **Figure 4C**). In addition, the AUC values of the 1-, 2-, and 3-year survival rates of the FAM-score prognostic model were 0.780, 0.910, and 0.942, respectively (**Figure 4E**). To further evaluate the prognostic ability of the FAM-score prognostic model, we calculated the FAM score in the test dataset (GSE87211). Similarly, patients with a low-risk score had a significantly favorable survival rate and DFS (p< 0.05, **Figures 4B, D**). The AUC values of the 1-, 2-, and 3-year survival rates in the test dataset were 0.736, 0.710, and 0.702, respectively (**Figure 4F**). Univariate and multivariate Cox regression analyses showed that the FAM score was an independent risk factor for DFS (both p< 0.05, **Figure 4G**).

In terms of OS, the same phenomenon was observed in both the training (GSE56699) and testing (GSE87211) datasets. The survival rates decreased with increasing risk scores (**Supplementary Figures 3A, B**). In the training cohort, OS was not significantly different between the high- and low-risk groups (p = 0.2, **Supplementary Figure 3C**). In the testing cohort, patients in the high-risk group had a worse OS than those in the low-risk group (p = 0.04, **Supplementary Figure 3D**). The AUC values of 1-, 2-, and 3-year overall survival rates in the training dataset were 0.933, 0.596, and 0.739, respectively, and in the testing dataset, they were 0.907, 0.868, and 0.864, respectively (**Supplementary Figures 3E, F**). Therefore, we considered that the FAM-score prognostic model has an excellent ability to predict DFS and OS in patients with rectal cancer who have undergone nCRT. Similarly, the FAM score was an independent risk factor for OS (p< 0.05, **Figure 4H**).

### Association between clinicopathological features and fatty acid metabolism-related signature

**Figure 5:**
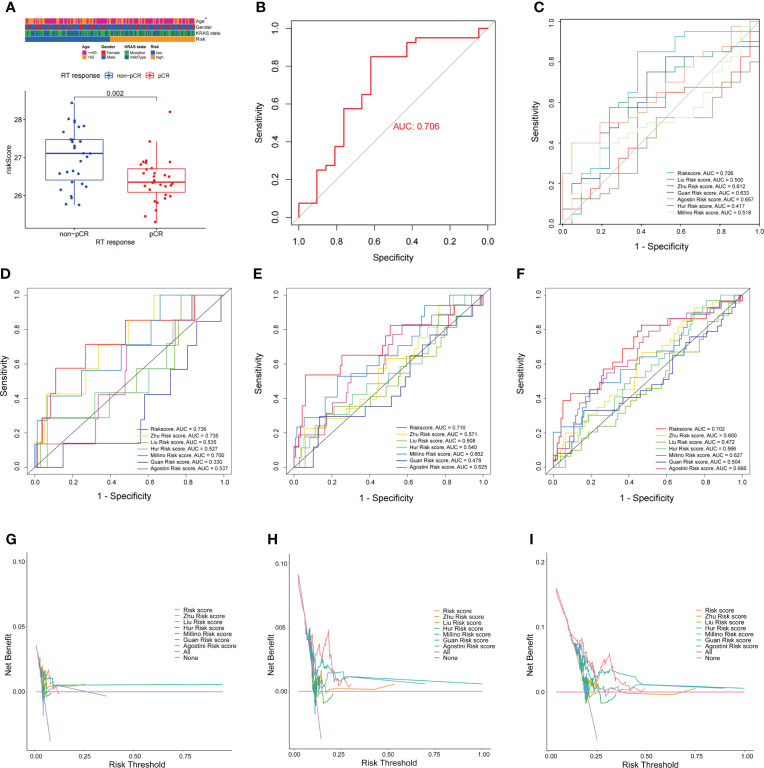
Comparison of FAM-related signature and previously published multi-gene models. **(A)** FAM-related risk score association with nCRT response. **(B)** ROC curves used to evaluate the ability of predicting nCRT response using FAM-related signature. **(C)** The ability of the FAM-score model to predict nCRT response of patients with rectal cancer treated with nCRT is better than previously published multi-gene models. **(D–F)** AUC values at 1-, 2-, and 3-years DFS of FAM-score model and previously published multi-gene models. **(G–I)** The DCA for FAM-score model compared with previously published multi-gene models. nCRT, neoadjuvant chemoradiotherapy; FAM, fatty acid metabolism; AUC, area under the curve; DCA, decision curve analysis.

FAM-related signatures were significantly associated with patient age (**Supplementary Figure 4A**). Patients who are less than 65 years of age had a higher FAM-related risk score than those older than 65 years (p = 0.039, **Supplementary Figure 4B**). In addition, patients who achieved pCR after nCRT had a lower risk score than nCRT-resistant patients (p = 0.002, **Figure 5A**). In predicting the nCRT responses of patients with rectal cancer, the FAM-related risk score showed promising predictive ability (AUC = 0.706, **Figure 5B**). Once more, our observations reflected the ability of the FAM score to predict nCRT response in patients with rectal cancer.

### Comparison of fatty acid metabolism-related signature and previously published multi-gene signatures

The FAM-related signature showed the potential ability to predict DFS, OS, and even nCRT response in patients with rectal cancer. To further evaluate the predictive ability of the FAM-related signature, we compared it with previously published multi-gene signatures. The FAM-related signature showed a better predictive ability for nCRT responses than the other previously published multi-gene signatures (**Figure 5C**). FAM-related signatures were more efficient in predicting the 1-, 2-, and 3-year DFS and OS of patients who received nCRT than other signatures (**Figures 5D–F**; **Supplementary Figures 5A–C**). Furthermore, the decision curve analysis (DCA) curves also suggested that the FAM-related signature outperformed the other signatures in predicting 1-, 2-, and 3-year DFS and OS (**Figures 5G–I**; **Supplementary Figures 5D–F**). Therefore, the FAM-related signature shows promising potential not only in predicting the prognosis of DFS and OS but also in predicting the nCRT response of patients with rectal cancer who underwent nCRT.

### Function enrichment of differentially expressed genes between high- and low-risk groups

**Figure 6:**
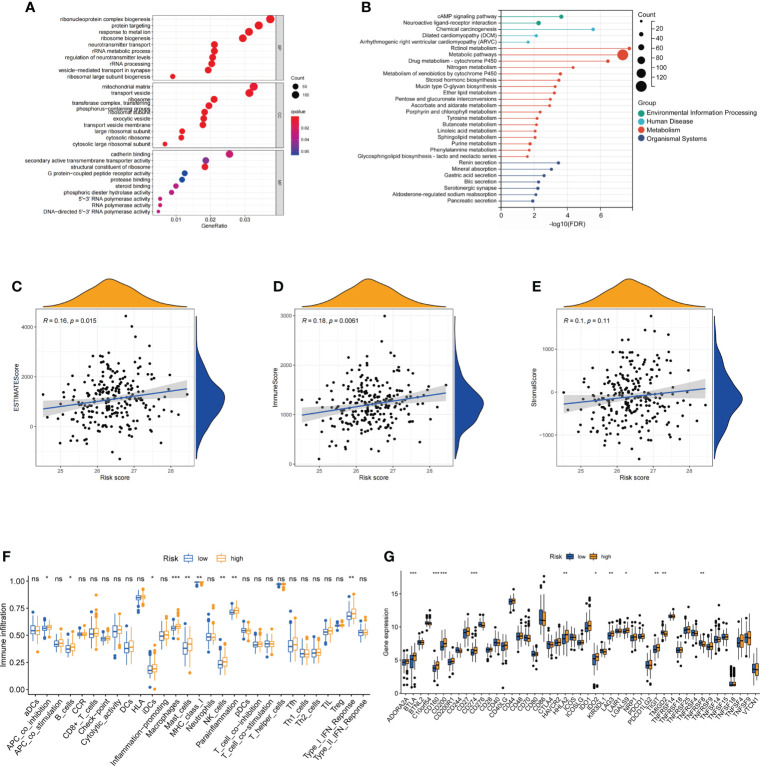
Functional analysis of differentially expressed genes and the immune landscape of fatty acid metabolism-related signatures. **(A, B)** The enriched GO and KEGG terms based on DEGs between low-risk and high-risk groups. **(C–E)** Correlation between estimate scores, immune scores, stromal scores, and risk scores. **(F)** The expression of immune checkpoints in low-risk and high-risk groups. **(G)** The ssGSEA score of immune cells and immune-related functions with low-risk and high-risk groups. GO, gene ontology; KEGG, Kyoto Encyclopedia of Genes and Genomes; DEGs, differentially expressed genes. ns: not significant; *: P< 0.05; **: P< 0.01; ***: P< 0.001.

GO and KEGG enrichment analyses were used to reveal the biological processes and functions of the DEGs between the high- and low-risk groups based on FAM-related signatures. GO enrichment analysis revealed that the functions of DEGs were enriched in the biogenesis and targeting of proteins, including the biogenesis of ribonucleoprotein complexes and calmodulin binding (**Figure 6A**). KEGG enrichment analysis revealed that the cAMP signaling pathway and neuroactive ligand–receptor interaction metabolic pathways were significantly enriched based on the DEGs between the two groups (**Figure 6B**).

### The landscape and evaluation of immune checkpoints based on fatty acid metabolism-related risk score

To further reveal the relationship between FAM-related signatures and tumor features, we first evaluated the tumor microenvironment (TME) score, including the estimated score, immune score, and stromal score, of patients with rectal cancer who underwent nCRT (**Figures 6C–E**). Patients with high FAM scores were closely associated with higher estimated and immune scores (both p< 0.05). Interestingly, we found that the number of immune-related cells, including interdigitating dendritic cells (iDCs), macrophages, and NK cells, was much higher in patients with a high FAM score (**Figure 6F**). We next analyzed the immune checkpoints to determine why patients with high-risk scores had higher immune cell infiltration but showed a worse treatment response. We observed that patients with high-risk scores were associated with high-level expression of genes associated with immune checkpoints, including *BTLA*, *CD160*, *CD200*, *CD274* (PD-L1), *HHLA2*, *IDO2*, *LAG3*, and *LGALS9* (p< 0.05, **Figure 6G**). These results also showed that patients with high-risk scores might respond better to therapies targeting the above checkpoints, especially PD-L1.

### Validation of the fatty acid metabolism-related signature in the Fujian Cancer Hospital cohort

**Figure 7:**
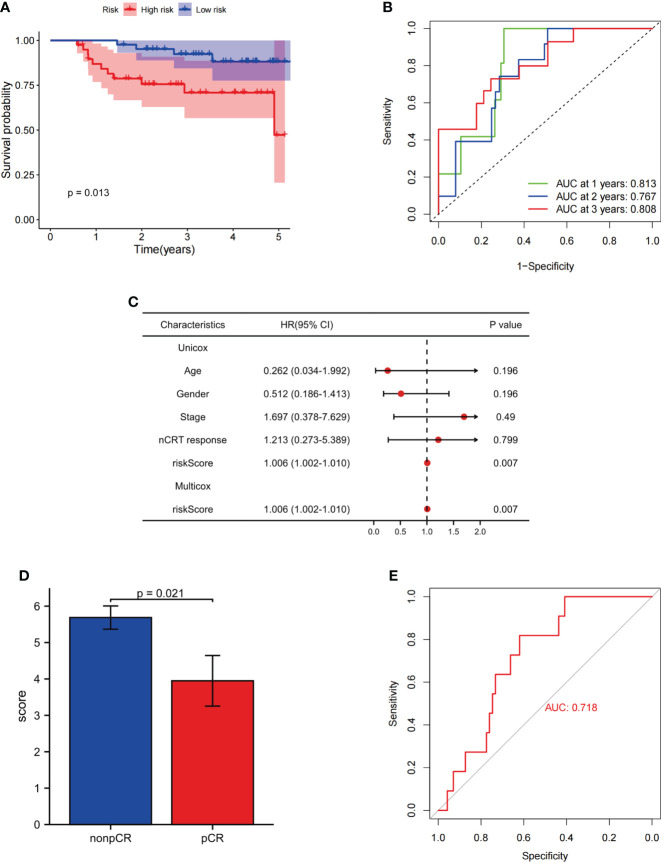
Evaluation of the ability of FAM-related signatures to predict prognosis and nCRT response in the FJCH set. **(A)** Kaplan–Meier survival analyses of DFS between patients with high-risk and low-risk scores in the independent cohort. **(B)** Time-dependent ROC curves used to evaluate the prognostic value of risk score. **(C)** Univariate Cox analysis and multivariate Cox analysis of clinicopathological features and FAM-related signature in DFS. **(D)** FAM-related risk score associated with nCRT response. **(E)** Evaluation of FAM-related signature to predict nCRT response. FAM, fatty acid metabolism; DFS, disease-free survival; OS, overall survival; ROC, receiver operating characteristic.

To confirm the ability of the FAM-related signature to predict DFS and OS, qPCR was used to examine the expression of five genes (*CYP1B1*, *DDC*, *ANO1*, *DAPL1*, and *RIOK3*) in 78 tumor tissues. The patients in our independent cohort were divided into high- and low-risk groups based on their FAM-related risk scores. Consistent with the training dataset, patients in the low-risk group had better DFS and OS rates (**Figure 7A**; **Supplementary Figure 6A**). The FAM-related signature also had the potential to predict DFS and OS based on ROC analysis (AUC values of 1-, 2-, and 3-year DFS rates were 0.813, 0.767, and 0.808, respectively; AUC values of 1-, 2-, and 3-year OS rates were 0.813, 0.735, and 0.762, respectively) (**Figure 7B**; **Supplementary Figure 6B**). Univariate and multivariate Cox regression analyses showed that the FAM score was an independent risk factor for DFS and OS (both p< 0.05, **Figure 7C**; **Supplementary Figure 6C**). In addition, patients with lower risk scores tend to achieve pCR in the FJCH cohort (p = 0.021, **Figure 7D**). The FAM-score model also showed a satisfactory ability to predict the response to nCRT (AUC = 0.718, **Figure 7E**).

## Discussion

Metabolism is considered a hallmark of cancer and is closely associated with tumor occurrence and development (28, 29). FAM has also been linked to cancer development and treatment response (30, 31). To the best of our knowledge, this is the first report revealing the important role of FAM in locally advanced rectal cancer that applies gut microbiome, metabolome, and human transcriptome sequencing based on the GEO database in patients with rectal cancer treated with nCRT. In addition, the FAM-related signature composed of five genes showed excellent ability not only in predicting DFS and OS but also in predicting nCRT response in patients with rectal cancer treated with nCRT.

At the baseline of patients with rectal cancer who underwent nCRT, we observed that the gut microbiome from the phylum Proteobacteria, including the class Betaproteobacteriales and the families Burkholderiaceae and Xanthobacteraceae, was strongly associated with favorable nCRT response. Proteobacteria are a major constituent of the human gut microbiome and are associated with the synthesis of medium-chain fatty acids and long-chain fatty acids (32–34). *Anaerotruncuscolihominis_DSM_17241*, which is also enriched in the pCR group of Ruminococcaceae, produces butyric and acetic acids (35, 36). Functional enrichment analysis based on the significantly different gut microbiomes also confirmed that linoleic acid metabolism, a part of FAM, was significantly different between patients in the pCR and non-pCR groups. Linolenic acid is inversely associated with the development of CRC (37). In addition, functional enrichment based on metabolism analysis showed a vital role of FAM in our study. FA intake has been associated with the occurrence of CRC (37, 38). Furthermore, peroxidase damage to polyunsaturated fatty acids drives ferroptosis, which is strongly related to radiotherapy-induced cell death (39, 40). Recently, targeting FAM has become a potential method for radiation sensitization of cancers (23, 41).

A previous study reported an association between FAM-related genes and CRC (41). Nevertheless, no similar multi-gene signature that can predict prognosis and treatment response has been developed based on FAM-related genes in patients with rectal cancer treated with nCRT. According to our results, previous models for predicting the prognosis of rectal cancer did not perform the same ability in such patients. In the current study, a FAM-related signature was constructed using *CYP1B1*, *ANO1*, *RIOK3*, *DDC*, and *DAPL1*, with favorable AUC at 1-, 2-, and 3-year DFS and 1-, 2-, and 3-year OS. In addition, it showed a high AUC for predicting nCRT response in patients with rectal cancer treated with nCRT. Cytochrome P450 1B1 (*CYP1B1*), a member of the cytochrome P450 (*CYP*) family, is highly expressed in tumor tissues, including in CRC, but its expression is lower than in normal tissues (42). A previous study confirmed a significant relationship between *CYP1B1* expression and poor prognosis in patients with CRC, which is similar to our result (43). *CYP1B1* is considered to be an important modulator of FAM and a potential therapeutic target in cancer therapy because of its ability to activate procarcinogens (44, 45). Anoctamin 1 (*ANO1*) is upregulated in CRC and is associated with cancer development by activating the mitogen-activated protein kinase signaling pathway (46, 47). Furthermore, *Fusobacterium nucleatum* has been found to promote the expression of *ANO1* in CRC cells, which prevents CRC cell apoptosis caused by chemotherapy (48). Right open reading frame kinase 3 (*RIOK3*) has been reported to be involved in cancer invasion and metastasis (49, 50). In addition, the expression level of *RIOK3* increases with hypoxia, which is an important factor in preventing effective radiotherapy and immunotherapy of cancer (51, 52). l-DOPA decarboxylase (*DDC*) has been proposed in recent research to serve apoptotic and antiproliferative functions with phosphatidylinositol 3-kinase (PI3K) (53, 54). Moreover, high expression of *DDC* is closely associated with better prognosis in CRC (55). Death-associated protein like-1 (*DAPL1*) has also been confirmed to be associated with cell death in previous studies (56, 57). In our study, *CYP1B1*, *ANO1*, and *RIOK3* were negatively associated with prognosis and nCRT response in rectal cancer. Nevertheless, *DDC* and *DAPL1* were positively associated with prognosis and treatment response in rectal cancer. This five-gene FAM-related model thus assists in predicting prognosis and guiding therapeutics in patients with rectal cancer treated with nCRT.

nCRT is undoubtedly an important treatment for LARC that can improve sphincter preservation and down-staging and decrease local recurrence. An increasing number of researches have shown that radioresistance is closely related to altered tumor metabolism (58, 59). Current evidence has confirmed the value of glycolytic metabolism in improving the sensitivity to radiation therapy for tumors (60), although the mechanism of fatty acid metabolism in improving the radiosensitivity of tumors is not clear. The extensive network of tumor metabolism is interconnected and plays an important role in affecting tumor radiosensitivity. Irradiation induces tumor cell death and can activate the immune response in the TME (61, 62). Interestingly, in this study, we observed that patients with high-risk scores had a higher abundance of immune cells and higher immune scores, which contradicted their higher immune cell infiltration and poor prognoses. However, further exploration of immune checkpoints yielded an explanation. We found that immune checkpoints showed higher expression levels of *CD274* (PD-L1) in patients with high-risk scores. Overexpressed PD-L1 in cancer cells binds to PD-1 on tumor-infiltrating lymphocytes with impaired T-cell activation (62, 63). In addition, some types of cells in the TME, including dendritic cells, also express PD-L1, which orchestrates the immunosuppressive microenvironment that supports tumor growth (64). Therefore, immune-related cells cannot act efficiently because of PD-L1 overexpression, although there is a higher abundance of immune cells. In addition, high expression levels of PD-L1 are associated with a poor prognosis of rectal cancer (65, 66). Currently, the PD-1/PD-L1 axis is considered an immunotherapeutic target for cancers (67). With intense research on immunotherapy, a combination of conventional cancer treatment methods with PD-L1 may benefit patients with rectal cancer (68). Thus, the overexpression of PD-L1 may provide a new therapeutic strategy for rectal cancer patients treated with nCRT.

Despite these promising results, some limitations remain. First, the FAM-related signature was constructed based on the GEO database and examined in an independent cohort; however, multicenter, large-scale prospective research is still needed to confirm the ability of FAM-related signatures to predict prognosis and nCRT response in patients treated with nCRT. Furthermore, this was a retrospective study, and clinical data were in some cases incomplete or unavailable, which caused a selection bias and incomplete analysis. The potential mechanism by which FAM impacts prognosis and treatment response in patients with rectal cancer needs to be explored. In the future, the results of the present study, including the microbiome, metabolism, and high-throughput sequencing, must be validated both *in vitro* and *in vivo*.

## Conclusion

In conclusion, FAM is an important link between the gut microbiome and treatment response. A novel FAM-related signature was constructed that has an excellent ability to predict prognosis in patients with rectal cancer treated with nCRT. In addition, the FAM-related risk score can also be recognized as a potential biomarker of nCRT response in such patients. Finally, the findings of this study provide innovative insights into the individualized management of patients with rectal cancer treated with nCRT.

## Data availability statement

The datasets presented in this study can be found in online repositories. The names of the repository/repositories and accession number(s) can be found below: NCBI under accession number PRJNA818503.

## Ethics statement

The studies involving human participants were reviewed and approved by The Ethics Committee of Fujian Cancer Hospital. The patients/participants provided their written informed consent to participate in this study.

## Author contributions

JL, JW, and HZ conceived and designed the study. YL, HL, and QW collected the samples. YX and YC recorded patient information. LK and GS performed bioinformatics analyses of the sequencing data. This manuscript was prepared by JL, JW, and HZ. All authors have contributed to the manuscript and approved the submitted version.

## Funding

This work was supported by a grant (no. 2021J01433) from the Natural Foundation of Fujian Province, Fujian Research and Training Grants for Young and Middle-aged Leaders in Healthcare, National Clinical Key Specialty Construction Program, 2021, and Startup Fund for scientific research, Fujian Medical University (grant number 2020QH2043).

## Acknowledgments

We thank the researchers and study participants for their contributions. We thank the TCGA and GEO databases for providing valuable and public datasets.

## Conflict of interest

The authors declare that the research was conducted in the absence of any commercial or financial relationships that could be construed as a potential conflict of interest.

## Publisher’s note

All claims expressed in this article are solely those of the authors and do not necessarily represent those of their affiliated organizations, or those of the publisher, the editors and the reviewers. Any product that may be evaluated in this article, or claim that may be made by its manufacturer, is not guaranteed or endorsed by the publisher.

## Abbreviations

ASV, amplicon sequence variants; AUC, area under the curve; DFS, disease-free survival; FAs, fatty acids; FAM, fatty acid metabolism; GEO, Gene Expression Omnibus; GO, Gene Ontology; KEGG, Kyoto Encyclopedia of Genes and Genomes; LARC, locally advanced rectal cancer; LASSO, least absolute shrinkage and selection operator; LDA, linear discriminant analysis; MSEA, metabolite set enrichment analysis; OS, overall survival; ROC, receiver operating characteristic; TRG, tumor regression grade; VIP, variable importance in projection.
