# Supplementary material for: Evaluation of the ability of fatty acid metabolism signature to predict response to neoadjuvant chemoradiotherapy and prognosis of patients with locally advanced rectal cancer
Source: Front Immunol. 2022 Nov 24;13:1050721. doi: 10.3389/fimmu.2022.1050721 (PMC9729334; doi:10.3389/fimmu.2022.1050721)
Supplement: Supplementary file 1 [file Image_1.pdf]

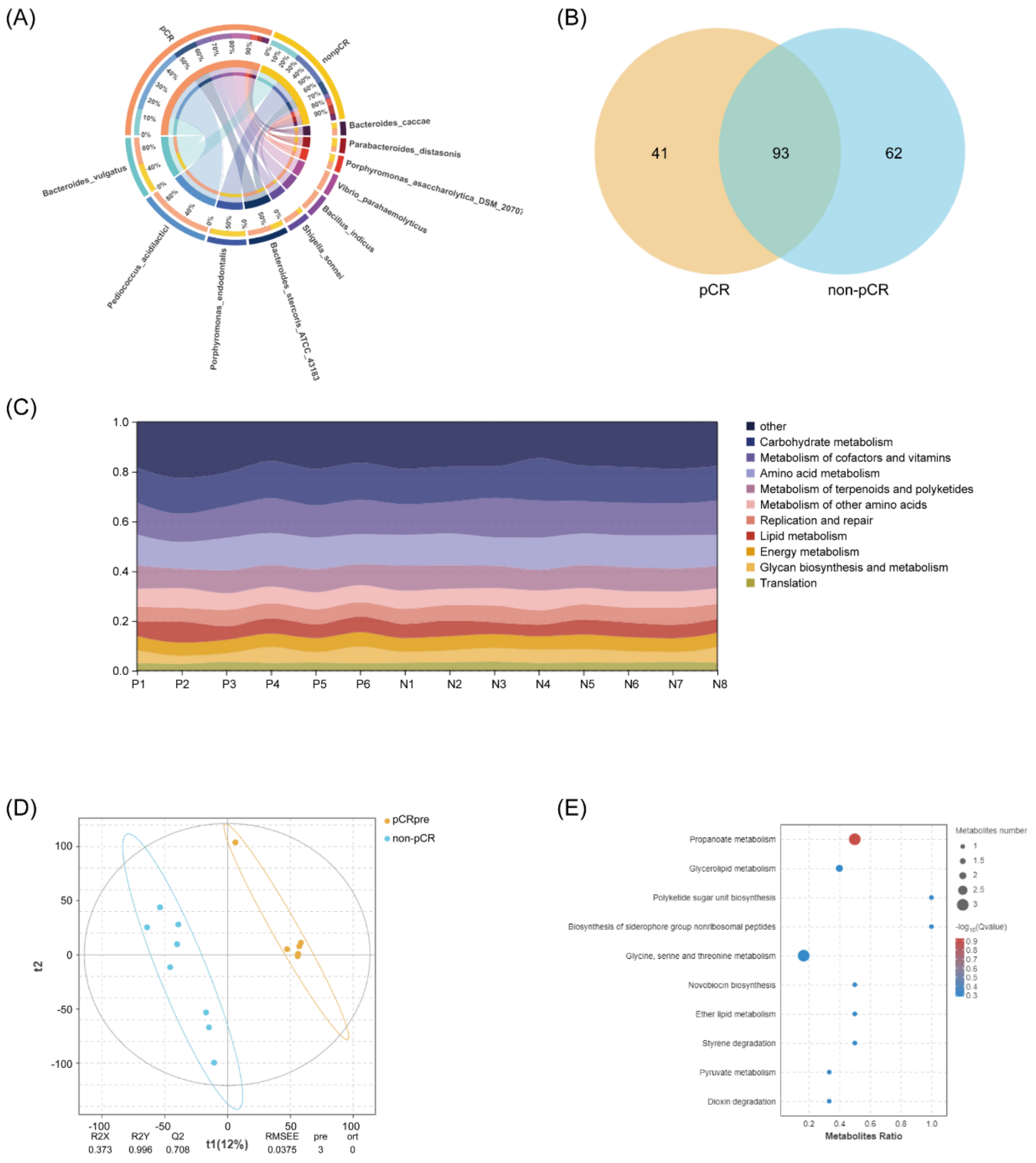

Supplementary Figure 1: Gut microbiome and metabolism characteristics at the baseline associated with neoadjuvant chemoradiotherapy treatment response. (A) The top 10 microbiomes of pCR- and non-pCR group. **(B)** Venn diagram in species level between patients in pCR group and non-pCR group. **(C)** Relative function of each patient (P: patients with pCR; N: patients with non-pCR). **(D)** Clustering of PLS-DA between pCR and non-pCR groups. **(E)** Significantly different signaling pathway between pCR group and non-pCR group ($p < 0.05$). **PLS-DA**: partial least-squares discriminant analysis; **pCR**: pathological complete response.

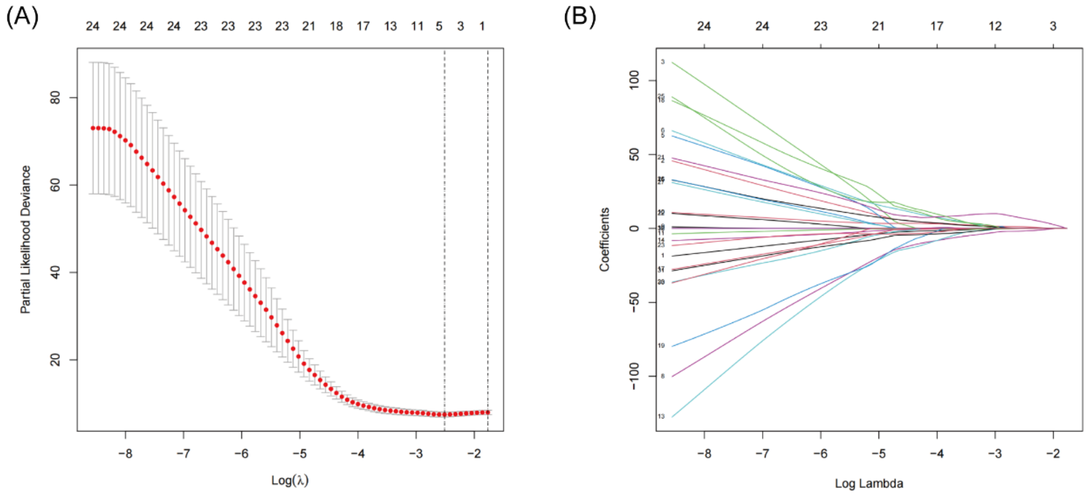

Supplementary Figure 2: Analysis of LASSO regression to screened the DEGs

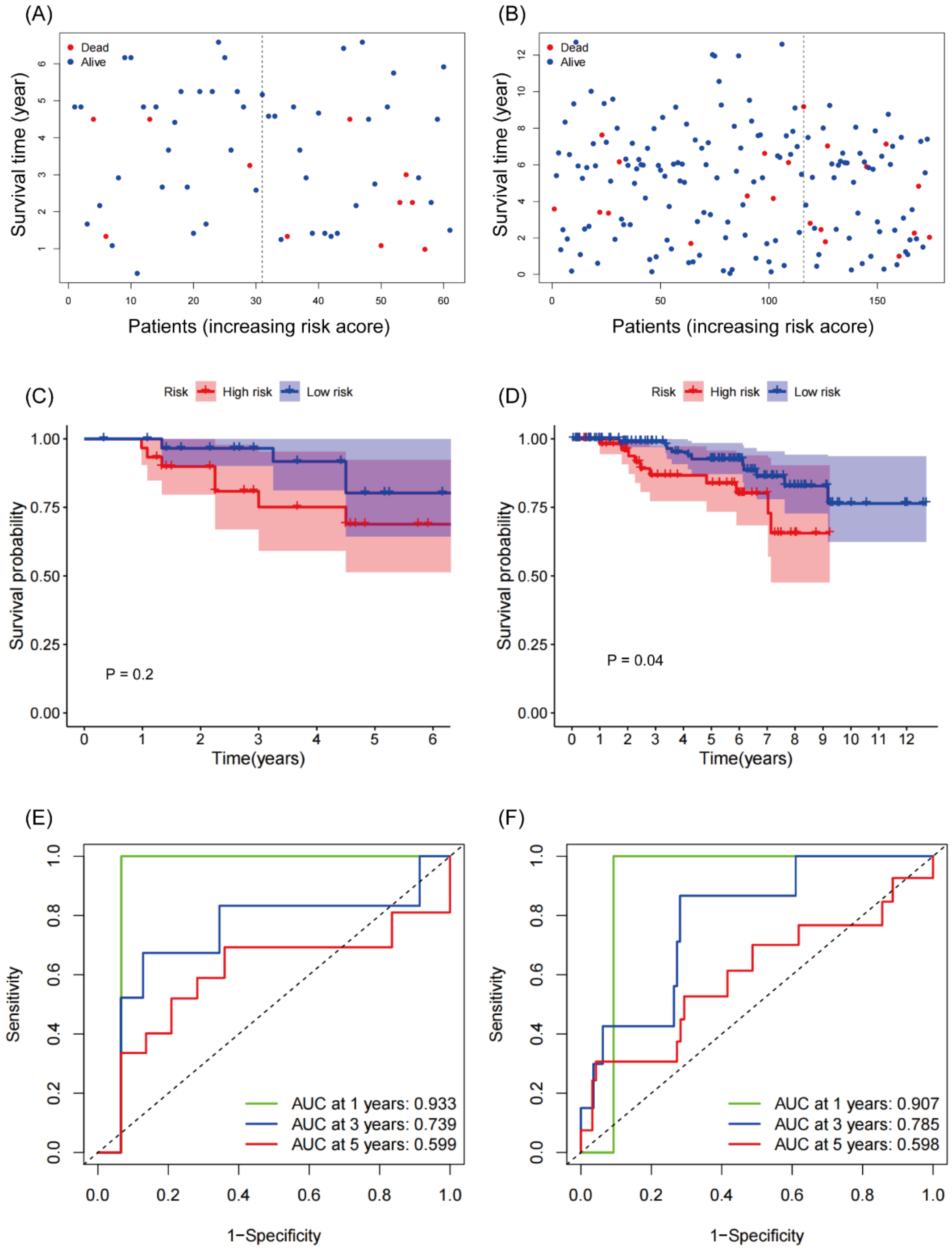

Supplementary Figure 3: Evaluation of the ability of fatty-acid-metabolism-related signature to predict prognosis in training and validation cohorts. (A-B) Association between OS and FAM-related risk score in the training cohort (GSE56699) and the validation cohort (GSE87211). **(C-D)** Kaplan-Meier survival analyses of OS between patients with high-risk and low-risk scores in the training and validation cohorts. **(E-F)** Time-dependent ROC curves used to evaluate the prognostic value of risk score in the training and validation cohorts. **FAM**: fatty acid metabolism; **OS**: overall survival; **ROC**: receiver operating characteristic.

(A)

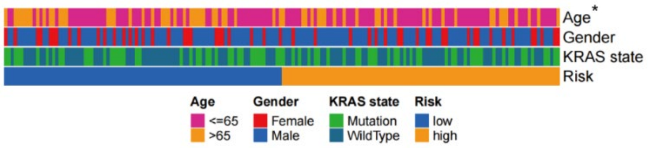

(B)

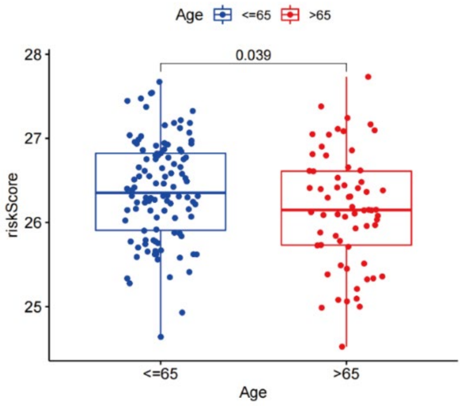

Supplementary Figure 4: Association between clinicopathologic features and fatty-acid-metabolism-related signature. (A) Relationship of FAM-related risk score and clinicopathologic features in patients with rectal cancer treated with nCRT. **(B)** Relationship of FAM-related risk score and age in patients with rectal cancer treated with nCRT. FAM: fatty acid metabolism.

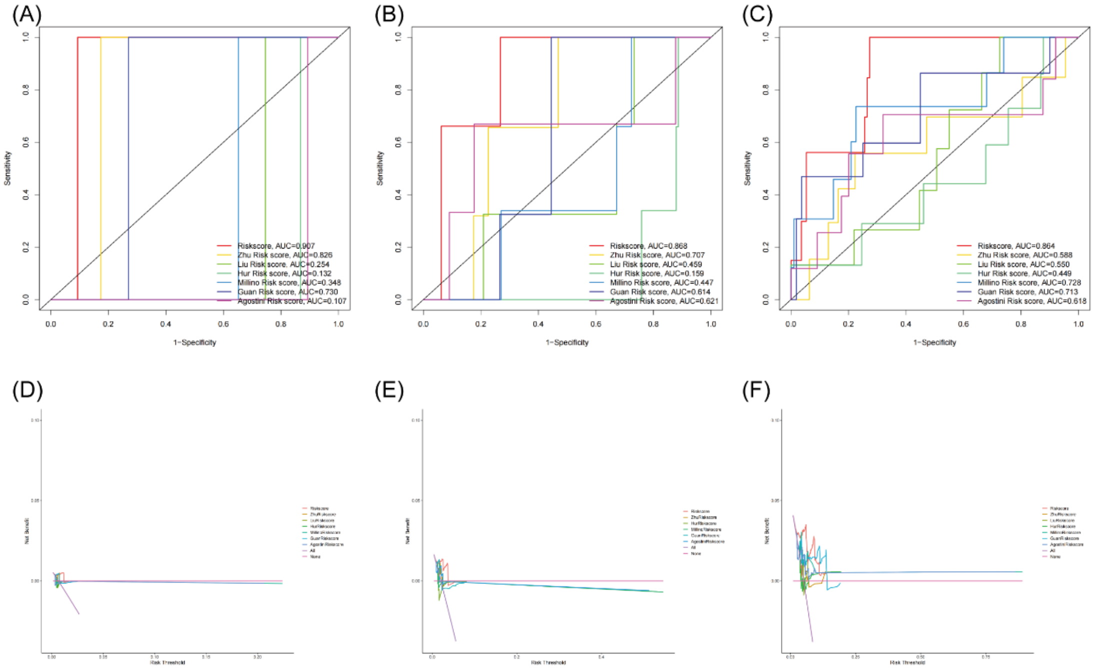

Supplementary Figure 5: Comparison of fatty-acid-metabolism-related signature and previously published multi-gene models. (A-C) The AUC values at 1-, 2-, 3-years OS of FAM-score model and previously published multi-gene models. **(D-F)** The DCA for FAM-score model compared with previously published multi-gene models. **nCRT**: neoadjuvant chemoradiotherapy; **FAM**: fatty acid metabolism; **AUC**: area under the curve.

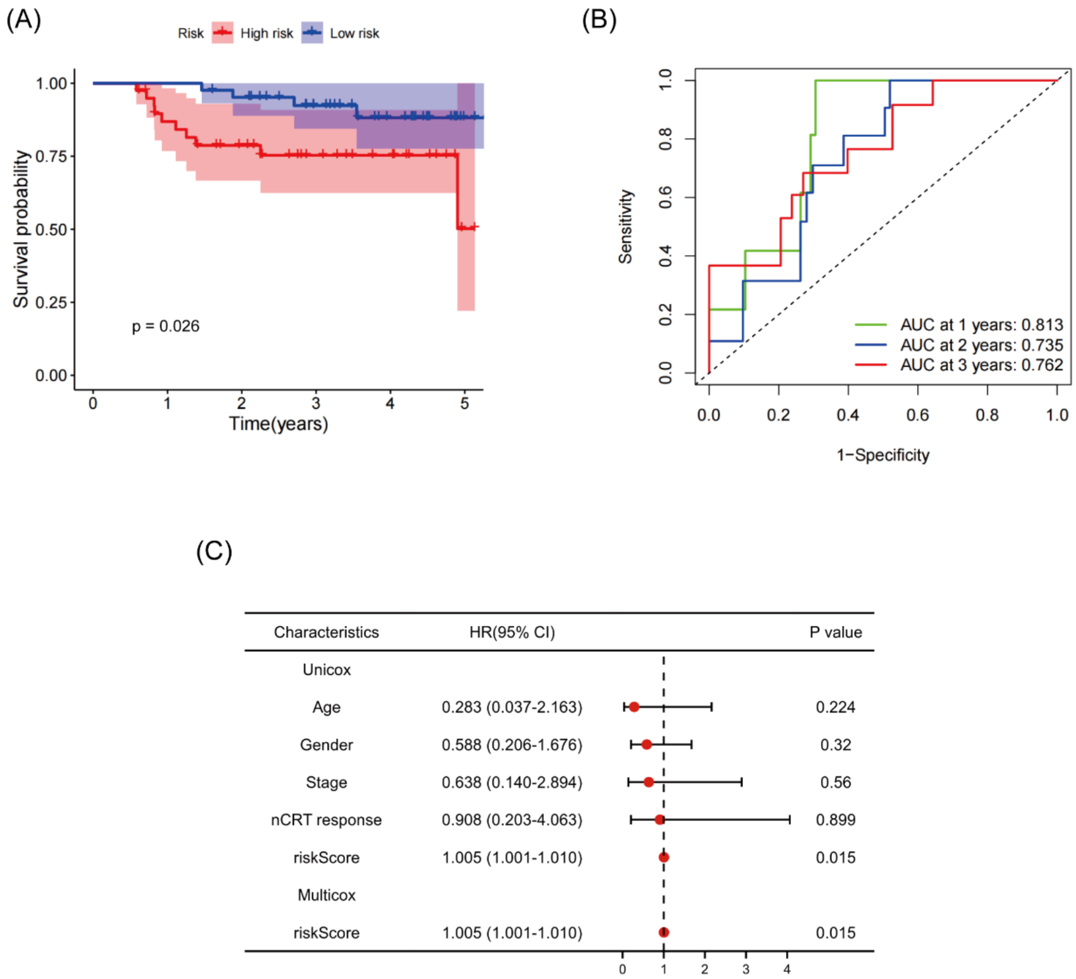

Supplementary Figure 6: Evaluation of the ability of FAM-related signatures to predict prognosis in the FJCH set. (A) Kaplan-Meier survival analyses of OS between patients with high-risk and low-risk scores in the independent cohort. **(B)** Time-dependent ROC curves used to evaluate the prognostic value of risk score. **(C)** Univariate Cox analysis and multivariate Cox analysis of clinicopathological features and FAM-related signature in OS. **FAM**: fatty acid metabolism; **OS**: overall survival; **ROC**: receiver operating characteristic.
